# Impact of higher order network structure on emergent cortical activity

**DOI:** 10.1162/netn_a_00124

**Published:** 2020-03-01

**Authors:** Max Nolte, Eyal Gal, Henry Markram, Michael W. Reimann

**Affiliations:** Blue Brain Project, École Polytechnique Fédérale de Lausanne, Geneva, Switzerland; Edmond and Lily Safra Center for Brain Sciences, The Hebrew University, Jerusalem, Israel; Department of Neurobiology, The Hebrew University, Jerusalem, Israel; Blue Brain Project, École Polytechnique Fédérale de Lausanne, Geneva, Switzerland; Laboratory of Neural Microcircuitry, Brain Mind Institute, École Polytechnique Fédérale de Lausanne, Lausanne, Switzerland; Blue Brain Project, École Polytechnique Fédérale de Lausanne, Geneva, Switzerland

**Keywords:** Neocortex, Microcircuit, Connectome, Morphology, Topology, Network, Model, Motifs

## Abstract

Synaptic connectivity between neocortical neurons is highly structured. The network structure of synaptic connectivity includes first-order properties that can be described by pairwise statistics, such as strengths of connections between different neuron types and distance-dependent connectivity, and higher order properties, such as an abundance of cliques of all-to-all connected neurons. The relative impact of first- and higher order structure on emergent cortical network activity is unknown. Here, we compare network structure and emergent activity in two neocortical microcircuit models with different synaptic connectivity. Both models have a similar first-order structure, but only one model includes higher order structure arising from morphological diversity within neuronal types. We find that such morphological diversity leads to more heterogeneous degree distributions, increases the number of cliques, and contributes to a small-world topology. The increase in higher order network structure is accompanied by more nuanced changes in neuronal firing patterns, such as an increased dependence of pairwise correlations on the positions of neurons in cliques. Our study shows that circuit models with very similar first-order structure of synaptic connectivity can have a drastically different higher order network structure, and suggests that the higher order structure imposed by morphological diversity within neuronal types has an impact on emergent cortical activity.

## Author Summary

We seek to understand how certain characteristics in the structure of neuron-to-neuron connectivity shape the activity of local neural circuits. The local connectivity of cortical networks features a nonrandom higher order structure characterized by the presence of tightly connected clusters of neurons. We use a biologically detailed model of neocortical microcircuitry that recreates these features and simplify its complexity algorithmically while preserving the larger scale connectivity trends. We then simulate spontaneous and evoked activity in the two models with simplified and complex connectivity and compare the resulting spiking statistics. The results allow us to characterize the role of the higher level structure of connectivity in interaction with other biological features shaping neuronal activity such as synaptic adaptation and noise.

## INTRODUCTION

Local synaptic connectivity between neocortical neurons is highly structured (Perin, Berger, & Markram, 2011; Song, Sjöström, Reigl, Nelson, & Chklovskii, 2005). Details of *first-order structure* that can be described by pairwise statistics include distinct mean connection strengths between different neuron types (Feldmeyer, Lubke, Silver, & Sakmann, 2002; Jiang et al., 2015; Le Bé, Silberberg, Wang, & Markram, 2007; Silberberg & Markram, 2007), distance-dependent connectivity that changes between neuron types (Fino & Yuste, 2011; Holmgren, Harkany, Svennenfors, & Zilberter, 2003; Jiang et al., 2015; Song et al., 2005), and a bias for reciprocal connections (Markram et al., 2015; Perin et al., 2011; Song et al., 2005). This first-order structure is undoubtedly important for emergent electrical activity, for example by constraining the interlaminar flow of spiking activity (Reyes-Puerta, Sun, Kim, Kilb, & Luhmann, 2014) and constraining the excitation-inhibition balance (Rosenbaum, Smith, Kohn, Rubin, & Doiron, 2017).

Yet, local synaptic connectivity also contains significant *higher order structure* that cannot be described by pairwise statistics (Benson, Gleich, & Leskovec, 2016). Examples are an overexpression of certain triplet motifs of neurons (Perin et al., 2011; Song et al., 2005) and an abundance of cliques of all-to-all connected neurons (Reimann, Nolte, et al., 2017). Such higher order structure has been hypothesized to be important for computation (Braitenberg, 1978; Hebb, 1949; Knoblauch, Palm, & Sommer, 2009; Willshaw, Buneman, & Longuet-Higgins, 1969). In recurrent spiking neural networks, clustering of neurons has been shown to impact dynamics (Litwin-Kumar & Doiron, 2012), and motifs of neurons have been shown to shape spike correlations (Bojanek, Zhu, & MacLean, 2019; Hu, Trousdale, Josi, & Shea-Brown, 2013; Recanatesi, Ocker, Buice, & Shea-Brown, 2019). On the other hand, modern artificial neural networks have demonstrated impressive computational capabilities without explicitly modeled, complex higher order microstructures (Simonyan & Zisserman, 2014). Whether computation in the cortex relies on higher order structure such as multineuron motifs on top of already complex first-order structure is unknown.

**Figure 1.**
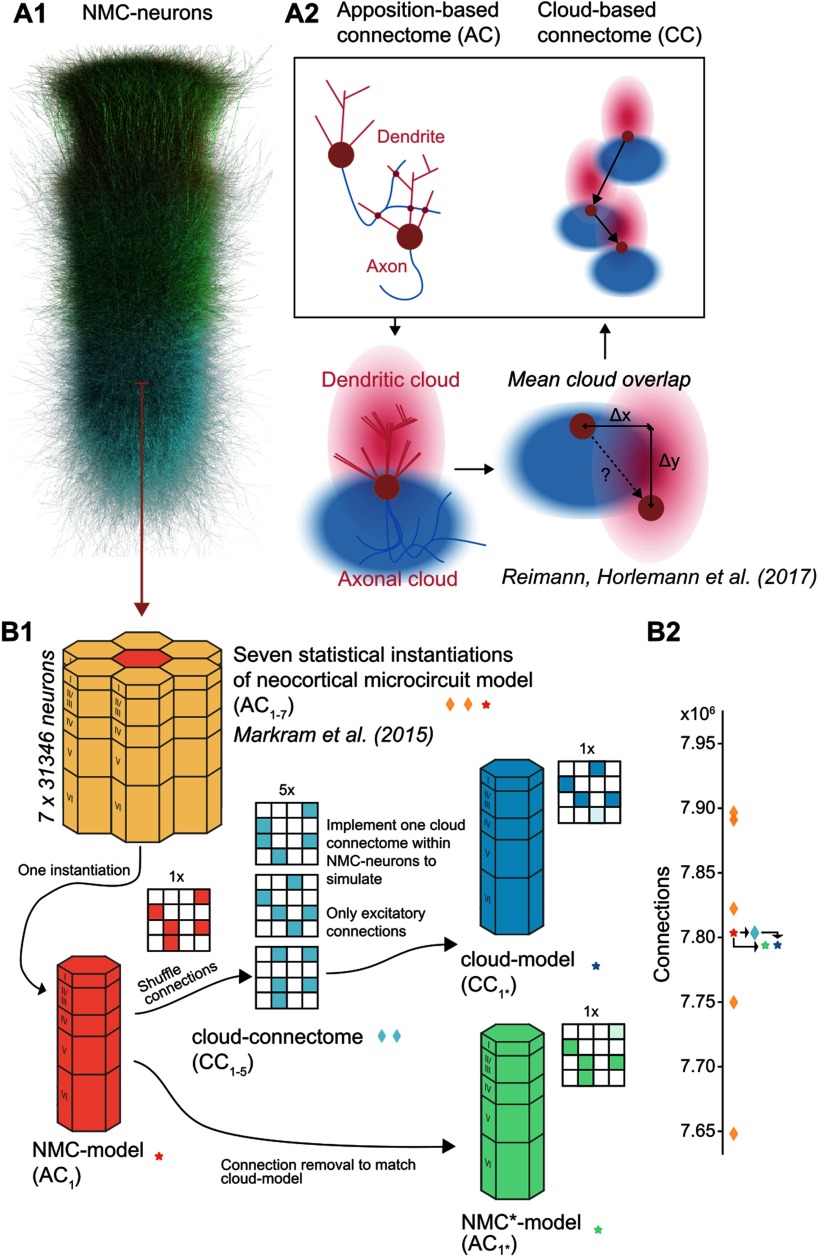
Reducing higher order network structure in a neocortical microcircuit model. (A1) Neurons in the neocortical microcircuit (NMC-) model. (A2) Deriving synaptic connectivity between neocortical neurons: Connectivity in the NMC-model is based on appositions of dendrites and axons (Reimann et al., 2015). Connectivity in the control cloud-model considers overlap of average dendritic and axonal clouds (Reimann, Horlemann, et al., 2017). (B1) We computed network properties for seven statistical instantiations of the microcircuit (apposition-based connectomes AC_1–7_, orange diamonds and red star; Markram et al., 2015), and simulated one of them in this study (the *NMC-model* with connectome AC_1_, red star). Additionally, we studied versions of the model using the existing NMC-neurons, but synaptic connectivity derived according to the cloud-based approach (cloud-connectomes CC_1–5_, blue diamonds). We then implemented one of the alternative connectomes within the existing synapses of the NMC-neurons, resulting in the *cloud-model* that we simulated, with connectome C_1*_ (blue star). The rewiring of the cloud-model was restricted to excitatory connections. (B2) Number of connections across connectomes. By design, the cloud-connectomes CC_1–5_ have the same number of connections as the NMC-model connectome AC_1_. However, the connectome implemented in the cloud-model CC_1*_ has 0.12% fewer connections than CC_1_ because of a mismatch in new connections and available synapses (see Figure 2A). To control for this loss, we generate a copy of the NMC-model with the same fraction of excitatory connections randomly removed, the NMC*-model (green star).

Answering this question in vivo will require simultaneous access to both detailed synaptic connectivity and electrical activity. While detailed synaptic connectivity of larger areas encompassing thousands of neurons might soon become available (Kasthuri et al., 2015; Yin et al., 2019), it will remain difficult to study the direct impact of the network structure on electrical activity, and even then it would be difficult to quantify the relative impact of first- and higher order structure. A modeling approach can help bridge this gap. An algorithmic approach was developed that uses available data to generate synaptic connectivity in a neocortical microcircuit model with diverse morphologies (Reimann, King, Muller, Ramaswamy, & Markram, 2015). When simulated, this neocortical microcircuit model (*NMC-model*, Figure 1A1) can reproduce an array of in vivo–like neuronal activity (Markram et al., 2015) and allow us to compare and manipulate detailed, predicted structure and function.

Here, we utilize a recent finding that first-order connectivity is largely constrained by morphological diversity *between* neuronal types, and higher order connectivity by morphological diversity *within* neuronal types (Reimann, Horlemann, Ramaswamy, Muller, & Markram, 2017). Both aspects are captured by the NMC-model, leading to a biologically realistic microstructure (Gal et al., 2017). By connecting neurons according to average axonal and dendritic morphologies (axonal and dendritic *clouds*, Figure 1A2), we create a control circuit (the *cloud-model*) that has very similar first–order structure, but reduced higher order structure. We find that this reduced higher order structure—caused by disregarding morphological diversity within neuronal types—includes fewer cliques, and decreased small-world topology. Additionally, the cloud-model is characterized by more homogeneous degree distributions with reduced in-degrees at the bottom of layer 6. When we simulate and compare the electrical activity in the two circuit models, we find that the changes in higher order connectivity are accompanied by nuanced changes in neuronal firing patterns and reduced topological ordering of pairwise correlations.

Our study reduces higher order network structure of a neocortical microcircuit model while leaving first-order structure largely intact, providing an alternative approach to cortical network studies that add increasingly complex structure to simple neural network models. Our approach ensures that the cortical network studied is closer to the biological ground truth than more simplifying models (albeit less amenable to theoretical analysis). Our comparison of reduced and nonreduced network structure suggests that higher order network topology of neocortical microcircuitry shapes cortical function.

## RESULTS

### Reducing Higher Order Network Structure in a Neocortical Microcircuit Model

The NMC-model consists of 31,346 neurons belonging to 55 different morphological types (*m-types*) (Figure 1A1). Synaptic connectivity between the neurons was derived by considering appositions of dendrites and axons as potential synapse locations (apposition-based connectome AC_1_, Figure 1A2, left), which were then filtered according to biological constraints (Reimann et al., 2015). While this connectome is merely a null model of connectivity, it matches a large array of biological measurements, in terms of both its *first-order structure* and its *higher order structure*. We define first-order structure as structure of synaptic connectivity that can be described by pairwise statistics, such as connection strengths between different m-types, distance-dependent connectivity, and average degrees, and higher order structure as structure involving more than two neurons (Benson et al., 2016), such as common-neighbor bias and overrepresentation of cliques of all-to-all connected neurons. Neuronal and synaptic physiology in the model are equally well constrained (Markram et al., 2015).

To assess the specific role of the higher order synaptic structure on neuronal activity, we had to reduce the higher order structure while simultaneously impacting the first-order structure as little as possible. To this end, we used an alternative cloud-based approach to derive synaptic connectivity, which is based on the overlap of average dendritic and axonal shapes of the various m-types (Figure 1A2, bottom, right; *cloud-model*) instead of specific axo-dendritic appositions of individual neurons as in the NMC-model. This approach (see Methods) has been shown to yield similar properties of first-order structure, such as distinct connection strengths between different m-types (and consequently between layers), distance-dependent connectivity, and a bias for reciprocal connections (Reimann, Horlemann, et al., 2017). However, the cloud-model cannot reproduce an experimentally observed bias for connected neocortical neurons to share a common neighbor (Perin et al., 2011; Reimann, Horlemann, et al., 2017), indicating a reduced complexity of its higher order structure. By comparing electrical activity between the NMC-model and the cloud-model in simulation experiments, we can study the relative impact of first- and higher order structure on electrical activity.

To build the cloud-model, we first generated alternative cloud-based connectomes for the NMC-neurons (Figure 1B1, red to green), using methods introduced by Reimann, Horlemann, et al. (2017). Briefly, average axon and dendrite shapes were calculated from reconstructions for all m-types. Next, for each combination of neuron types, their axon and dendrite volumes were convolved (see Figure 1A2) to yield the expected strength of their overlap for all possible relative soma locations. Soma locations of neurons were taken from the NMC-model and used to look up the overlap strengths for all neuron pairs. Selection probabilities for all pairs were then calculated as proportional to the square of the overlap. For each pair of neuron types, we randomly picked according to those probabilities a number of connections that were equal to the number of connections between them in the NMC-model (random picking without replacement; Figure 1B2, red asterisk and green diamond).

**Figure 2.**
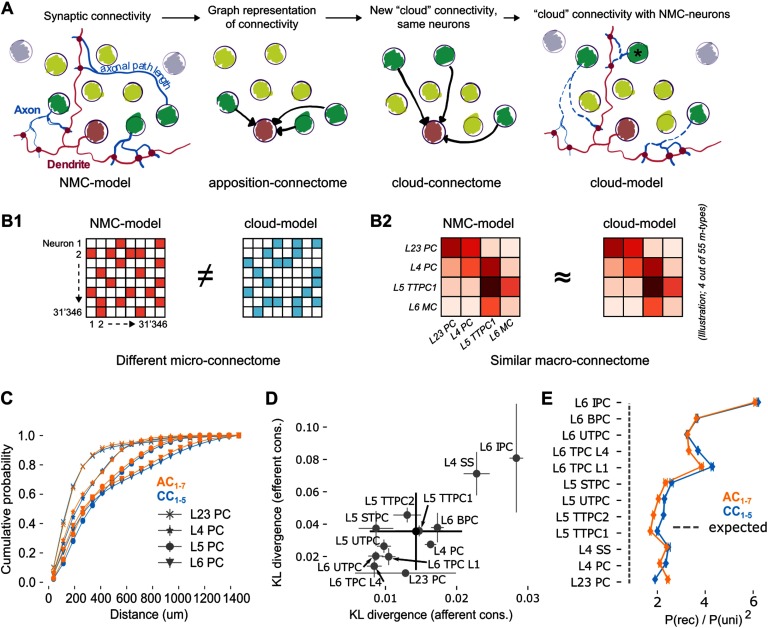
Rewiring synaptic connectivity in a neocortical microcircuit model preserves macroconnectivity trends. (A) Left: In the NMC-model, synaptic connections from a given morphological type (*m-type*; neurons in light/dark green) are based on axo-dendritic appositions. Synaptic delays are calculated from *axonal path lengths*. Center left: The apposition connectome for the m-type can be represented as a directed graph that excludes neurons from other m-types. Center right: A new, cloud-based connectome is calculated for the same neuron population. Right: The cloud-based connectome is implemented by rewiring existing synapses, preserving their dendritic locations and physiological parameters. The process also preserves their original synaptic delay even for neurons at a different distance than the original neuron (black asterisk). (B) The NMC- and cloud-models have a completely different microconnectome in terms of connections between individual neurons (B1) but a very similar macroconnectome in terms of number of connections between the 55 different m-types in the model (B2). (C) Soma-to-soma distance for connected neurons pairs where the postsynaptic neuron is in the indicated layer (distance of afferent connections) for AC_1–7_ (orange) and CC_1–5_ (blue). (D) Kullback-Leibler divergence of afferent and efferent connection distances between AC_1_ and CC_1–5_. Gray circles and error bars indicate mean and standard deviation over instances for all excitatory m-types. Black: overall mean and standard deviation over five instances. (E) Reciprocal overexpression in terms of reciprocal connection probability divided by the square of the unidirectional connection probabilities for all excitatory m-types. Orange: AC_1–7_, blue: CC_1–5_. Indicated are mean and standard deviation.

A neuron-to-neuron connection matrix was then instantiated from the probabilities (the *cloud-connectome*, CC_1–5_) and transplanted into the NMC-neurons, to generate an instance of the *cloud-model* that was identical to the NMC-model in terms of neuronal composition, and morphology and physiology of all individual neurons (Figure 1B1). Similarly, the physiology of individual synapses (strength, kinetics, and short-term dynamics) and their locations on dendrites were taken from the NMC-model—we only changed which presynaptic neurons innervated them when implementing the cloud-based connection matrix (Figure 2A). This reassignment of innervation was constrained to select a new innervating neuron only from the same m-type that innervated it in the NMC-model to preserve the m-type-specific synaptic physiology. However, while synaptic physiology is guaranteed to be conserved in this approach, the axonal *path length*, and thus the time it takes for an action potential to propagate from the soma to the synapse, is potentially biologically implausible. While the average path lengths per pre-/postsynaptic m-type combination are conserved, an action potential might potentially arrive earlier or later than is appropriate for the distance between pre- and postsynaptic neuron (see Figure 2A). To verify that the average distance-dependence of connectivity is conserved, we considered the probability distribution of distances of connected neurons for individual layers (Figure 2C). We calculated the Kullback-Leibler divergence of this distribution between NMC-model and cloud-connectome, both for afferent and efferent connections (Figure 2D). The divergence for individual m-types ranged from 0.008 to 0.029 with an overall mean of 0.014 for afferent connections and from 0.016 to 0.08 with a mean of 0.036 for efferent connections, much lower than the divergence between the NMC-model and an Erdös-Rényi (ER) model that preserves in- or out-degrees (1.304 ± 0.661 for afferent connections and preserving in-degree; 0.749 ± 0.433 efferent, preserving out-degree; not shown). Similarly, we validated that the overexpression of reciprocal connectivity is preserved (Figure 2E).

In the cloud-based connection matrix CC_1_, a small number of neurons receive input from m-types that do not innervate them in the NMC-model. Consequently, a small fraction of connections could not be instantiated within the existing synapses of the NMC-neurons and had to be left out. The loss was minor for excitatory connections (0.12% loss of connections) but posed a significant problem for inhibitory connections. We therefore implemented the cloud-connectome only for excitatory connections and kept inhibitory connectivity in the cloud-model identical to the NMC-model, yielding a hybrid cloud-model connectome CC_1*_. Supplementary Figure S1 provides an overview of connection losses in the cloud-model. However, note that the loss of connections is very small compared with the variability in connections between statistical instantiations of the NMC-model (Figure 1B2, orange diamonds), which all have very similar dynamical properties (Markram et al., 2015).

To control for the minor loss of excitatory connectivity and the shuffling of axonal path lengths, we generated an additional control circuit, the NMC*-model (Figure 1B1). A total of 0.12% of excitatory connections were randomly removed from the NMC-model to match the m-type-specific connection losses in the cloud-model (as in Supplementary Figure S1B). The axonal path length parameter was then shuffled within connections with the same presynaptic excitatory m-type to account for a similar scrambling in the cloud-model (see Figure 2A). All circuit models and connectomes analyzed in this study are summarized in Table 1.

**Table 1.** Overview of circuit models and connectomes analyzed in this study

**Name**	**Description**
**NMC-neurons**	31,346 neuron models of a neocortical microcircuit (Markram et al., 2015).
**AC_1_**	Apposition-based connectome for NMC-neurons (Markram et al., 2015).^*a*^
**NMC-model**	Combination of NMC-neurons and AC_1_ (Markram et al., 2015).
**AC_2–7_**	Apposition-based connectomes of statistical variants of NMC-model (Markram et al., 2015).^*a*^
**CC_1–5_**	Cloud-based alternatives to AC_1_ (Reimann, Horlemann, et al., 2017).
**Cloud-model**	Combination of NMC-neurons and CC_1_ (excitatory connections) and AC_1_ (inhibitory connections).
CC_1*_	Connectome of cloud-model (see above).
NMC*-model	NMC-model with axon path length shuffle and 0.12% exc. connection loss as in cloud-model.

a mc2_Column and mc[0, 1, 3-6]_Column respectively at bbp.epfl.ch/nmc-portal/downloads → AVERAGE.

In summary, our approach ensures that for each neuron in the NMC-model, there is a corresponding neuron in the cloud-model with identical location, morphology, electrophysiology, synaptic physiology, inhibitory innervation, and average excitatory innervation patterns. On a larger scale, both models consequently have nearly identical *macroconnectomes* in terms of the number of connections between morphological types (Figure 2B2), and thus also between layers, and between excitatory and inhibitory subpopulations (Supplementary Figure S1C1–3). Only the microconnectomes defined by connections between individual neurons were changed within tight global constraints (Figure 2B1). An overview of what is conserved between NMC-model, cloud-model, and NMC*-model can be found in Table 2.

**Table 2.** Overview of control model conservation of NMC-model properties

**Microcircuit properties**	Cloud-model	NMC*-model	
Specific neuron electrophysiology & morphology	✓	✓	By design: neuron models reused; Figure 1
M-type-specific synaptic physiology	✓	✓	Synapses reused; connections shuffled only within m-type; Figure 2A
Average (pre-/postsynaptic m-type-specific) axonal path length	✓	✓	Reused synapses include path length parameter; Figure 2A
Macroconnectome (between m-types)	(✓)^*a*^	(✓)^*a*^	Figures 1B2, 2CDE
Microconnectome (between specific) neurons	x	(✓)^*a*^	Figures 1B1, 2A, 2B1, 3
Specific axonal path length	x	x	Figure 2A

a 0.12 excitatory connection loss.

### Decreased Heterogeneity of Degree Distributions in the Cloud-Model

**Figure 3.**
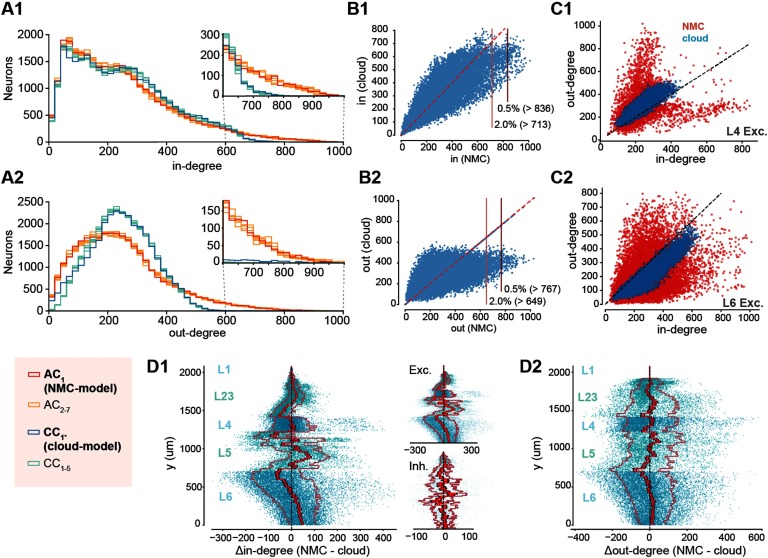
Decreased heterogeneity of degree distributions in cloud-model. (A1) In-degree distributions of neurons in NMC-model (AC_1_, red), in additional apposition-based connectomes (AC_2–7_, orange), the cloud-model (CC_1*_, blue), and the five cloud-connectomes (CC_1–5_, light blue). Insets show the same distributions starting from 600 for easier comparison. (A2) As A1, but for out-degree distributions. (B) Hub neurons in NMC- and cloud-models. (B1) Scatterplot of in-degree in NMC- versus cloud-model for the same neurons. Horizontal lines indicate 2.0% and 0.5% percentile in NMC-model. (B2) As B1 but for out-degree. (C1) In- versus out-degree scatterplot for NMC- and cloud-model for excitatory neurons in layer 4. (C2) In- versus out-degree scatterplot for NMC- and cloud-model for excitatory neurons in layer 6. (D1) Difference in in-degree of neurons between NMC- and cloud-model, across cortical depth. The bright red line indicates the mean across y-bins, the dark red line the standard error of the mean, and the outer, faded line the standard deviation. Insets: for excitatory and inhibitory subpopulations. (D2) As D1, but for out-degree.

By design, the average in- and out-degree of neurons belonging to a specific m-type was preserved in the cloud-connectome. However, the distribution around the mean was altered. Degree distributions have been demonstrated to shape cortical network dynamics (Landau, Egger, Dercksen, Oberlaender, & Sompolinsky, 2016), with long-tailed distributions being associated with highly structured brain networks (Gal et al., 2017). We can see that both in- and out-degree distributions are more heterogeneous in the NMC- than in the cloud-model (Figure 3A1 and 3A2, blue vs. red), which applies to all layers (Supplementary Figure S2), and is also preserved when connection strength in the form of maximum synaptic conductance is taken into account (Figure S3). In the NMC-model, the standard deviation of out-degrees (*σ*_*out*_(AC_1_) = 152, statistical variants: *σ*_*out*_(AC_2–7_) = 150–154 [min − max]) and the standard deviation of in-degrees (*σ*_*in*_(AC_1_) = 176, *σ*_*in*_(AC_2–7_) = 173–179) are higher than in the cloud-model (*σ*_*out*_(CC_1*_) = 110, cloud-connectomes: *σ*_*out*_(CC_1–5_) = 101.7–101.8; *σ*_*in*_(CC_1*_) =158, *σ*_*in*_(CC_1–5_) = 158.2–158.4). The discrepancy in *σ*_*out*_ and *σ*_*in*_ of CC_1–5_ and AC_1–7_ is significantly different (*σ*_*out*_: *p* = 3.0 × 10^−14^, *σ*_*in*_: *p* = 1.6 × 10^−8^; two-sided *t* test), although both are almost one order of magnitude over the value expected in an Erdös-Rényi (ER) network of the same size and sparseness (*σ*(*ER*) = 15.7).

The stark difference in connectivity between the NMC- and cloud-models is also reflected by hub neurons, previously defined as the top 0.5% of neurons in terms of in- or out-degree (Gal et al., 2017), which vanish in the cloud-model when using the same cutoff value as in the NMC-model (Figure 3B). We further found a redistribution of connectivity in terms of in- and out-degree from the bottom to the top of layer 6 (Figure 3D1 and 3D2). Finally, differences in degree distributions extended to higher order properties in the form of correlations between in- and out-degree (Figure 3C1 and 3C2 for excitatory neurons in layers 4 and 6; see Supplementary Figure S4 for all neurons), indicating a stronger specialization into input and output neurons in the NMC-model. In summary, the cloud-model has a strongly reduced heterogeneity of connectivity in terms of distributions of in- and out-degrees.

### Decreased Small-Worldness and Fewer Directed Simplices in Cloud-Model

**Figure 4.**
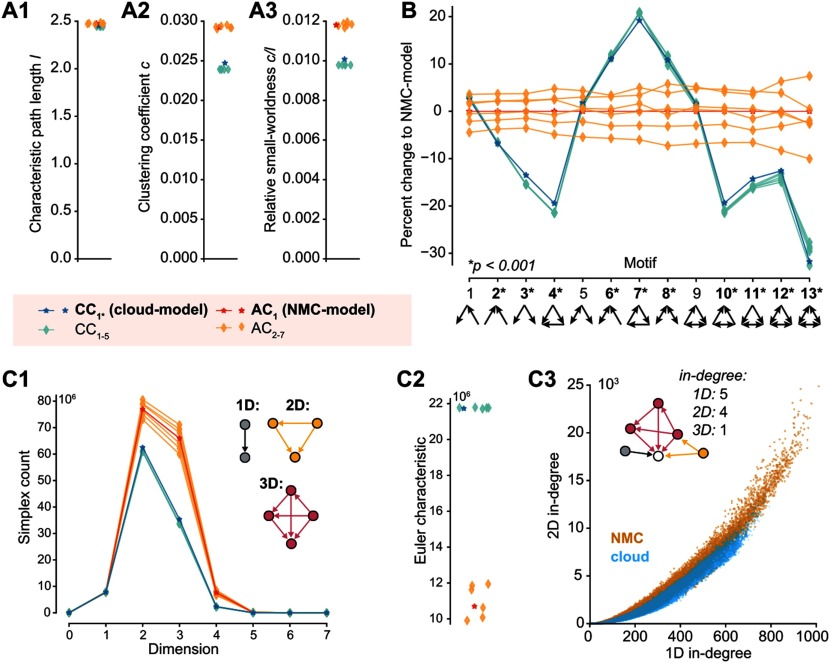
Decreased small-worldness and fewer directed simplices in cloud-model. (A1) Characteristic path length *l* of the different connectomes. (A2) Clustering coefficient *c* of the different connectomes. (A3) Relative small-worldness *c*/*l* of the different connectomes. (B) Percentage change in total number of all triplet motifs from NMC-model (*p* values: *t* test between AC_1–7_ and CC_1–5_). (C1) Number of directed cliques (simplices) per dimension in the different connectomes (for zoom in see Figure S5A). (C2) Euler characteristic (alternating sum of number of simplices). (C3) Participation at the sink of 1D simplices (standard in-degree) versus 2D simplices (2D in-degree: the number of simplices a neuron is part of as the sink of a simplex).

The higher order structure of the NMC-model results, for example, in a bias for two connected neurons to share common neighbors (*common-neighbor bias*), a tendency that has been demonstrated to be reduced in the cloud-model (Reimann, Horlemann, et al., 2017). It also manifests itself on a global scale in the form of a small-world network topology (Gal et al., 2017). A network is considered small-world if its global clustering coefficient is considerably larger than that of an ER network of the same size and sparseness, while the characteristic path length is roughly equal to that of the ER network. For networks of the same size and sparseness, the ratio of the global clustering coefficient *c* and the characteristic path length *l* provides a measure of relative small-worldness. The clustering coefficient describes the probability of neurons that share a common neighbor to be directly connected, a tendency that has previously been shown to be reduced in the cloud-model (Reimann, Horlemann, et al., 2017). Consequently, we find that the cloud-model has a reduced clustering coefficient (*c* is around 20% larger in the NMC-model than in the cloud-model, Figure 4A2). The characteristic path lengths *l* of NMC- and cloud-models are, however, almost equal to each other (Figure 4A1) and to the one of the equivalent ER network (around 2.15). Both models can therefore be considered small-world networks, although this tendency is significantly stronger in the NMC-model than in the cloud-model (Figure 4A3).

As an increased clustering coefficient indicates the tendency to form tightly connected motifs, we next compared the numbers of specific triplet motifs in both models (Figure 4B). Interestingly, the cloud-model has a decrease of motifs with forward transitive connectivity (Figure 4B, motif 4), but an increase in motifs with backwards transitive connectivity, such as cycles (motif 7). We previously showed that the NMC-model contains an abundance of a specific class of motifs called directed simplices (Reimann, Nolte, et al., 2017). These simplices generalize the forward transitive connectivity of motif 4 to motifs of any size. The size of a simplex is then called its dimension, defined as its size minus 1. While simplices of the same dimension are present in cloud- and NMC-models (Supplementary Figure S5A), the number of simplices in a given dimension is much higher in the NMC- than in the cloud-model (Figure 4C1). At the same time, the cloud-model is much closer to the NMC-model than simpler control models: In a previously used control that conserves only the distance-dependence of connectivity, but ignores the shapes of axonal and dendritic clouds, we found a more drastic decrease from around 80 million to 40 million 2D-simplices (Reimann, Nolte, et al., 2017), while the cloud-model has more than 60 million 2D-simplices. A distinct Euler characteristic (Figure 4C2) and distinct Betti numbers (Supplementary Figure S5B) further illustrate the change in global properties of network topology (see Methods). The increase in simplex numbers in the NMC-model follows from the more heterogeneous degree distributions, as neurons with larger degrees are generally part of more simplices (Figure 4C3).

In summary, the cloud-model—with its disregard for morphological diversity within neuronal types—has reduced small-worldness and reduced numbers of high-dimensional, forward transitive motifs.

### Impact of Higher Order Structure on Spontaneous Activity

**Figure 5.**
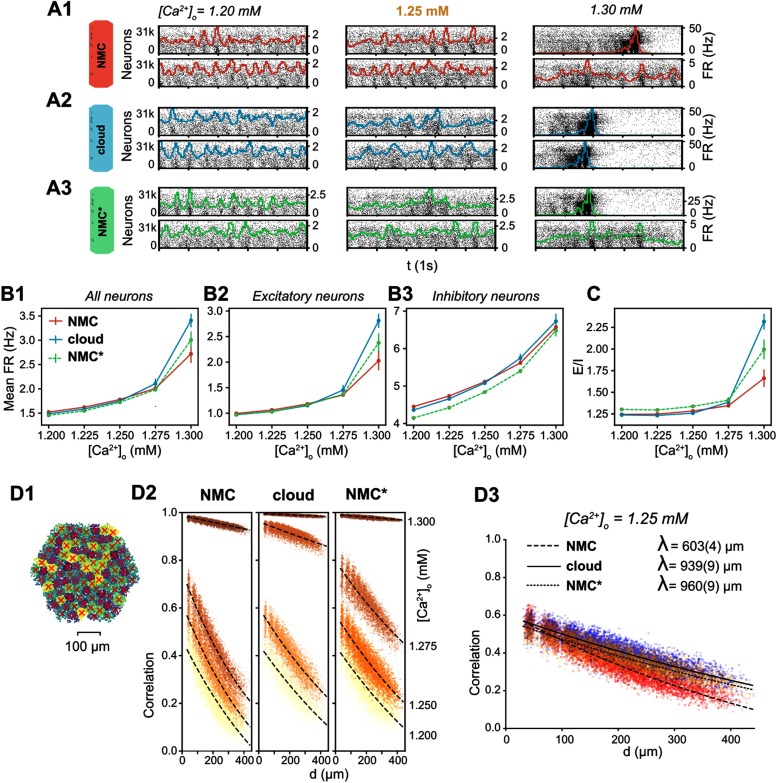
Simulating spontaneous activity in NMC- and cloud-models. (A1) Spontaneous activity of all neurons in the NMC-model for two trials at different levels of [*Ca*^2+^]_*o*_. Each spike is represented by a vertical line, whose position on the y-axis is ordered by soma position in the microcircuit, which was then rasterized. Colored lines depict the population firing rate (Δ*t* = 10 ms). (A2) Spontaneous activity of all neurons in the cloud-model. (A3) Spontaneous activity of all neurons in the control NMC*-model. (B1) Mean firing rate during spontaneous activity for all neurons. Mean of 20 trials of 1,000 ms, error bars indicate standard error of the mean. (B2) Mean firing rate during spontaneous activity for excitatory neurons. Mean of 20 trials of 1,000 ms, error bars indicate standard error of the mean. (B3) Mean firing rate during spontaneous activity for inhibitory neurons. Mean of 20 trials of 1,000 ms, error bars indicate standard error of the mean. (C) Total spike count of excitatory neurons divided by the spike count of inhibitory neurons. Mean of 20 trials of 1,000 ms, error bars indicate standard error of the mean. (D1) All neurons in the microcircuit were divided into 100 interlaminar clusters (k-means clustering). The red cross marks the geographic center of all neurons for each cluster. (D2) Correlation coefficients between combined firing rate histograms of all neurons of each combination of clusters versus distance between clusters. From [*Ca*^2+^]_*o*_ = 1.200 mM (bottom fitted curve, bright yellow dots) to [*Ca*^2+^]_*o*_ = 1.300 mM (top fitted curve, dark brown dots). The fitted line indicates an exponential fit *e*^−*d*/*λ*^ + *c*. (D3) As in D2, but at [*Ca*^2+^]_*o*_ = 1.25 mM. The fitted line indicates an exponential fit *e*^−*d*/*λ*^ + *c*, parentheses indicate the standard error of the fit.

To study the impact of the higher order structural differences on emergent activity, we next simulated spontaneous activity in NMC-, cloud-, and NMC*-models in an in vivo–like state, in which the NMC-model can reproduce several properties of cortical activity (Markram et al., 2015). We compared firing rates in this in vivo–like state (Figure 5B, [*Ca*^2+^]_*o*_ = 1.25 mM). Interestingly, excitatory firing rates are conserved (Figure 5B2), while inhibitory firing in the NMC*-model is significantly reduced compared with the cloud-model and NMC-model (Figure 5B3). That is, a small decrease of inhibitory firing rates due to the axonal path length shuffle and loss of E-to-I synapses in the NMC*-model is reversed by the reduced higher order structure of the cloud-model, reaching inhibitory firing rates similar to the NMC-model. This implies that the loss of higher order structure in the cloud-model leads to a shift towards more inhibition (Figure 5C).

However, the functional excitation-inhibition balance also depends on extracellular calcium levels ([*Ca*^2+^]_*o*_), which differentially modulates the release probability of excitatory and inhibitory synapses (Markram et al., 2015). In the NMC-model, at low [*Ca*^2+^]_*o*_, the network is in an asynchronous state of activity with lower firing rates (Figure 5A1, [*Ca*^2+^]_*o*_ = 1.2 mM, 1.25 mM). At high [*Ca*^2+^]_*o*_, the circuit is in a nonbiological synchronous state of activity with spontaneous network bursts and higher firing rates (Figure 5A1, [*Ca*^2+^]_*o*_ = 1.3 mM,1.35 mM). At [*Ca*^2+^]_*o*_ = 1.25 mM, just before the transition from the asynchronous to synchronous state, activity in the microcircuit is in the in vivo–like state described above.

The cloud-model appears to transition at lower [*Ca*^2+^]_*o*_ than the NMC-model and NMC*-model, with more rapidly increasing firing rates for higher [*Ca*^2+^]_*o*_ (Figure 5B1–3), an indication that effective excitation is stronger in the cloud-model than in NMC- and NMC*-models. [*Ca*^2+^]_*o*_ also regulates distance-dependent correlation coefficients of spiking activity between neurons (Figure 5D1; Markram et al., 2015). At the transition from asynchronous to synchronous activity, correlation coefficients rapidly increase (Figure 5D2). We can see that at [*Ca*^2+^]_*o*_ = 1.25 mM, correlations between NMC- and NMC*-model are very similar, but the cloud-model is in fact slightly ahead, with higher correlation coefficients (Figure 5D2). At that level of [*Ca*^2+^]_*o*_, correlations also drop slightly faster with distance in the cloud- than in the NMC-model; however, this is fully explained by the nontopological changes controlled for in the NMC*-model (Figure 5D3).

We thus conclude that the higher order network structure has a superficial impact on emergent population dynamics during spontaneous activity, such as a small increase in inhibitory firing rates, and paradoxically, a small increase in effective global excitation.

### Impact of Higher Order Structure on Evoked Activity

**Figure 6.**
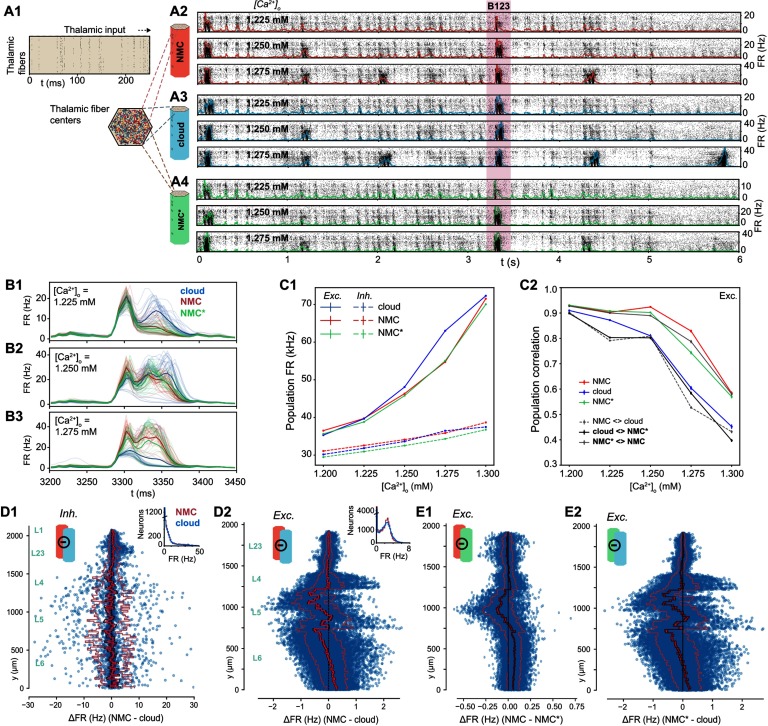
Simulating evoked activity in NMC- and cloud-models. (A1) Illustration of the first 250 ms of the thalamic input stimulating the microcircuit from *t* = 0 to 5 s. (A2) Evoked activity for all neurons in the NMC-model in response to input from A1 at three different [*Ca*^2+^]_*o*_-levels. (A3) Evoked activity for all neurons in the cloud-model at three different [*Ca*^2+^]_*o*_-levels. (A4) Evoked activity for all neurons in the NMC*-model at three different [*Ca*^2+^]_*o*_-levels. (B1) Time-dependent population firing rate for NMC-model (red), cloud-model (blue), and NMC*-model (green) for a 250-ms time period during the evoked activity (shaded red area in A234) at [*Ca*^2+^]_*o*_ = 1.225 mM. Faint lines: population means for all 30 trials. Thick line: mean of all 30 trials. Bin size: Δ*t* = 5 ms. (B2) As B1, but for [*Ca*^2+^]_*o*_ = 1.25 mM. (B3) As B1 and B2, but for [*Ca*^2+^]_*o*_ = 1.275 mM. (C1) Mean population firing rate of excitatory and inhibitory subpopulations during evoked activity at different [*Ca*^2+^]_*o*_-levels. Error bars indicate standard error of the mean over 30 trials. Note that error bars are smaller than linewidth. (C2) Average correlation coefficient between excitatory population PSTHs (Δ*t* = 5 ms) for 30 trials. Mean of 30 × (30 − 1)/2 = 435 combinations for same model, and mean of 30 × (30 + 1)/2 = 465 combinations between models. (D1) Difference in firing rate of inhibitory neurons during evoked activity at [*Ca*^2+^]_*o*_ = 1.25 mM. Blue dots indicate values for individual neurons, ordered along their soma positions with respect to the y-axis (cortical depth). Lines indicate mean (bright red), standard error (dark red), and standard deviation. Inset: Distribution of all mean firing rates. (D2) As D1, but for excitatory neurons. (E1) As D2, but for NMC-model and NMC*-model. (E2) As D1, but for NMC*-model and cloud-model.

Spontaneous activity in the NMC-model is variable and chaotic, yet thalamic stimuli can evoke highly reliable responses (Nolte, Reimann, King, Markram, & Muller, 2019). To study the impact of higher order structure on this evoked activity, we next stimulated the NMC- and cloud-models with thalamic input (Figure 6A1; see Methods). Similar to spontaneous activity, the response of the circuits to the input depended on [*Ca*^2+^]_*o*_ (Figure 6A2–4). As we established that the NMC- and cloud-models have a slightly different transition between dynamic states, we compared evoked activity for up to five [*Ca*^2+^]_*o*_ values around the in vivo–like state. All three models exhibited similar fluctuations of the overall firing rate at various [*Ca*^2+^]_*o*_ levels, responding robustly with brief increases in firing to periods of correlated thalamic input (Figure 6A2–4). As before, mean firing rates are very similar between the models, but the cloud-model has increased excitation, especially for larger [*Ca*^2+^]_*o*_ (Figure 6C1, Supplementary Figure S6A1).

To gain a more detailed understanding of the difference, we next looked at the changes in firing rates for individual neurons. Excitatory firing rates varied according to the change in in-degree across cortical depth (Figure 6D2 vs. Figure 3D1). The mean change directly reflects the change in in-degree shown in Figure 3D1—which is not surprising as any change in in-degree in the cloud-model is restricted to excitatory connections. While both excitatory and inhibitory neurons respond to the change in in-degree individually (Supplementary Figure S6B), the systematic shift in in-degree emerges only for excitatory, but not for inhibitory, neurons (see insets in Figure 3D1).

Time-dependent response patterns differ between models and [*Ca*^2+^]_*o*_-levels (Figure 6B1–3), with distinct patterns and increased trial-by-trial variability in the cloud-model when compared to activity in NMC-models and NMC*-models. To quantify this difference, we calculated the correlation coefficients of the peristimulus time histograms (PSTHs) between individual trials of the same model and different models (Figure 6C2, Supplementary Figure S6A2). The correlation between trials of the same model—that is, the reliability of the population response—generally decreased with increasing [*Ca*^2+^]_*o*_-level, although for the NMC- and NMC*-models it remained over 0.9 until 1.25 mM and was significantly higher than for the cloud-model at all levels. Correlations between different models were highest between NMC- and NMC*-models, further reinforcing that the slight loss of excitatory connections alone does not explain the observed changes in response patterns of the cloud-model.

We have previously shown that the in-degree also influences the spike-time reliability (*r*_spike_) of individual neurons in response to repeated trials of a thalamic stimulus (Nolte et al., 2019). While there is overall little change in *r*_spike_ going from NMC- to cloud-model (Supplementary Figure S7ABC), we observed a drop in reliability near the top of layer 5, which also displays a large in-degree reduction in the cloud-model. Further, as neurons with reduced in-degree towards the bottom of layer 6 spike less, they also become less reliable (Supplementary Figure S7BD). Similar to the firing rate, the change in spike-time reliability is clearly correlated with the change in in-degree (Supplementary Figure S7E).

### Impact on Ordering of Correlations in Simplices

We have shown that the cloud-model has a reduced bias for forward transitive triplet motifs (Figure 4B), resulting in a reduced number of directed simplices (Figure 4C1). We have further demonstrated that this leads to changes in the activity patterns of the circuit, specifically a reduced reliability of the population response to thalamic input. It has been shown that reliable responses are linked to increased correlations of synaptic inputs (Nolte et al., 2019; Wang, Spencer, Fellous, & Sejnowski, 2010), and we previously observed that such input correlations can be generated by directed simplex motifs with stronger correlations found in larger simplices (Reimann, Nolte, et al., 2017).

**Figure 7.**
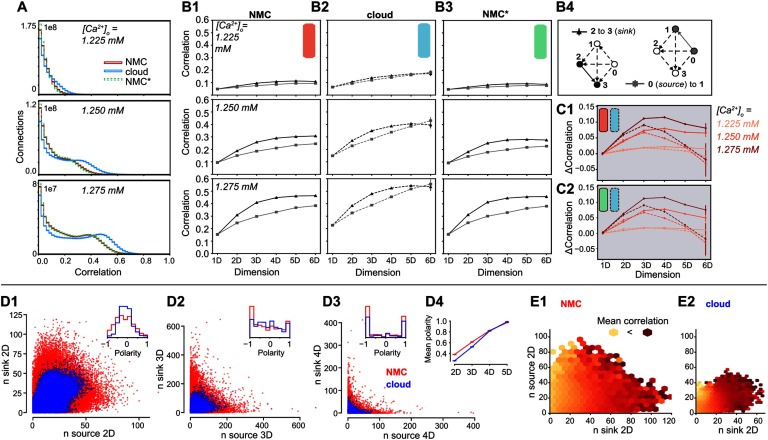
Simplices and correlations in NMC- and cloud-models. (A1) Correlation coefficients of the firing rates (Δ*t* = 20 ms) of all connected pairs of active neurons in the microcircuit for NMC-model, cloud-model, and NMC*-model, during the 30 trials of the thalamic stimulus (shown: ≥ 0). (B1) Average correlation coefficient of a connected pair of neurons in a (maximal) simplex of a certain dimension, depending on the position of the pair in the simplex; for the NMC-model, for three different [*Ca*^2+^]_*o*_-levels. Black triangles indicate the average correlation for a pair of neurons at the *sink* of a simplex, gray squares the average correlation for a pair at the *source* of a simplex. (B2) As B1, but for cloud-model. (B3) As B1, but for NMC*-model. (C1) Difference in average correlation coefficient for connections of neurons at the sink and the source of a (maximal) simplex. Solid lines: NMC-model; dashed lines: cloud-model. (C2) As C1, but for NMC*-model (solid lines) and cloud- model (dashed lines). (D1) Participation of connections in the source versus sink of (nonmaximal) 2D-simplices in NMC- and cloud-models (constrained to connections at the center of layers 4, 5, 6). Inset: Polarity is defined as $\frac{n_{\text{source}}-n_{\text{sink}}}{n_{\text{source}}+n_{\text{sink}}}$. (D2) As D1, but for 3D-simplices. (D3) As D1, but for 4D-simplices. (D4) Mean polarity for each dimension, defined as mean of $\frac{\left|n_{\text{source}}-n_{\text{sink}}\right|}{n_{\text{source}}+n_{\text{sink}}}$. (E1) Mean correlation given the participation of a connection in (nonmaximal) 2D-simplices at the source and sink, from bright yellow (low correlation) to dark red (high correlation); in NMC-model, constrained to connections at the center of layers 4, 5, 6. (E2) As D1, but for the cloud-model.

We therefore analyzed the structure of spike-time correlations of pairs of neurons in simplices across models (Figure 7A). As before (see Figure 5D), the overall strengths of correlations were comparable, though slightly higher in the cloud-model at identical [*Ca*^2+^]_*o*_-levels. This effect can be explained by the shift along the spectrum from asynchronous to synchronous activity (see above). However, we observed a qualitative difference in the local structure of correlations within a simplex. As a directed structure, each simplex can be uniquely sorted from the *source* neuron, with only outgoing connections to all other neurons in the simplex, to the *sink* neuron, with only incoming connections (Figure 7B4). While the correlations increased with simplex size for all three models (Figure 7B1–3), for the NMC- and NMC*-models they strongly depended on the location of neurons in the simplex, with highest correlations for the pair at the sink and lowest correlations for the pair at the source. Conversely, in the cloud-model this difference was reduced and disappeared for the largest simplex sizes (Figure 7C).

In other words, correlations in simplices have a hierarchical organization that leads to a specialization between input (source) neurons and output (sink) neurons that is diminished in the cloud-model. This is consistent with the earlier finding that longer-tailed degree distributions in the NMC-model allow the formation of specialized input- and output-neurons on a structural level (see Figure 3C). In fact, this effect extends from the in- and out-degree of individual neurons to an equivalent measure of simplex participation of connections. For each connection, we count the number of simplices in which it forms the first connection at the source, equivalent to the out-degree, and the number of simplices in which it forms the last connection at the sink, equivalent to the in-degree. As before, we found higher degrees in the NMC-model (Figure 7D1–3). Two effects led to higher degrees with the same number of connections. First, in the higher order structure of the NMC-model a larger number of simplices were formed (Figure 4C1); secondly, lower correlations between in- and out-degree in the NMC-model led to a polarization into connections used predominantly at the sink or source of many simplices. We quantified this *polarity* of a connection as the relative difference between its in- and out-degree with respect to simplices of a given dimension. We found that its absolute value increased with simplex dimension and was overall higher in the NMC-model, indicating a stronger structural polarization (Figure 7D4).

Taken together, we hypothesize that each simplex provides correlated input to neuron pairs at its sink, which in turn leads to correlated firing of that pair. While the effect of a single simplex on a pair may be negligible, strong structural polarization into inputs and outputs suggests that such pairs are likely to participate as the sink of an unexpectedly high number of simplices, thus dramatically increasing the size of the effect. The increased number of high-dimensional simplices and stronger polarization of the NMC-model allows some connections to participate in even more simplices as the sink. This hypothesis predicts that the correlation of a neuron pair is determined by the in-degree of its connections and is unaffected by its out-degree. We therefore quantified how the spike correlations of neuron pairs depend on these measures (Figure 7E). Indeed, we found a strong dependence on the in- but not the out-degree for both models.

## DISCUSSION

We introduced a method to reduce the higher order structure of synaptic connectivity in a neocortical microcircuit model, based on a previously published control connectome with a reduced common-neighbor bias (Reimann, Horlemann, et al., 2017)—the so-called cloud-model. In this cloud-model, excitatory synaptic connectivity between neurons was derived from average morphologies rather than appositions of axons and dendrites of individual neurons (Figure 1). By design, this approach preserved neuronal and synaptic physiology and locations, as well as the aggregate connection strength between m-types. We further demonstrated that it also preserved macroconnectomic trends, such as distance-dependent and reciprocal connectivity. We showed that the microconnectome of the cloud-model displayed systematic changes indicating a reduced higher order structure, such as fewer all-to-all directed cliques of neurons and reduced small-worldness (Figure 4), but also more homogeneous in- and out-degree distributions and the disappearance of hub neurons (Figure 3).

Spontaneous and evoked dynamics in the NMC-model and cloud-model are superficially very similar (Figures 5–6), a result that is not surprising given the conserved first-order structure, including conserved interlaminar connectivity, structural excitation-inhibition balance, and distance-dependent connectivity. However, some properties of neuronal activity changed. Spike counts of individual neurons changed according to in-degree, and population responses were shifted towards more excitation, with less reliable population responses (Figure 6). Most importantly, a hierarchical dependence of correlation strength on the position of a pair of neurons in directed motifs (simplices) was weaker in the cloud-model than in the NMC-model (Figure 7). We have demonstrated that this was a result of the more homogeneous in- and out-degree distributions leading to a reduced polarization into input- and output-neurons, and consequently reduced participation in simplices.

While the cloud-model has reduced higher order structure, it also has altered degree distributions. The differentiation between conserved first-order structure and reduced higher order structure is thus not as clean as we might have hoped. Nevertheless, reducing higher order network structure while keeping degree distributions fixed is possible, and such higher order structure can have an impact on network dynamics (Ritchie, Berthouze, House, & Kiss, 2014). To better understand the respective impact of changes in in- and out-degrees on one side, and clustering and high-dimensional motifs on the other side, it will be necessary to create a more refined control model that conserves degree distributions on top of first-order structure. An alternative derivation of the cloud-connectome could select connections from overlap strengths such that the in- or out-degree of individual neurons in the NMC-model is preserved, keeping the degree distributions identical. However this comes at the cost of no longer strictly conserving the number of connections between pairs of neuron types, thus fundamentally altering the first-order structure of the circuit.

The comparison between NMC- and cloud-models comes with several caveats. The preservation of distance-dependent connectivity in the cloud-model is better for some m-types than for others (Figure 2D). Distance-dependance is however much better conserved than in previous control models that disregarded the average shapes of m-types, resulting in many fewer simplices (Reimann, Nolte, et al., 2017). The cloud-model further neglects to reduce inhibitory higher order structure. However, the structural similarity between cloud-connectomes (changed excitatory and inhibitory connectivity) and the cloud-model (changed excitatory connectivity only) suggests that the contribution of inhibitory connections to higher order network structure in the NMC-model is negligible. This could potentially be due to an underestimation of inhibitory higher order structure in the NMC-model, either because of insufficient biological data constraining the connectivity, or because inhibitory structure might only emerge through plasticity (Vogels, Sprekeler, Zenke, Clopath, & Gerstner, 2011).

Indeed, this brings us to the most important caveat: The NMC-model is a statistical reconstruction of a prototypical microcircuit from sparse data. While it captures a high level of detail of synaptic connectivity (Gal et al., 2017), with strong constraints on the space of connectivity that can be explored by structural plasticity (Reimann, Horlemann, et al., 2017), the model has not learned to respond to specific stimuli or perform certain computations. A comparison to numbers of simplices observed in in vitro slice experiments shows that the number of simplices in the NMC-model is likely underestimated by an order of magnitude (Reimann, Nolte, et al., 2017). This suggests that a large fraction of biological higher order structure is not captured by the NMC-model. At the same time, learning mechanisms have been shown to lead to biological structural features, such as overexpression of triplet motifs (Zhang, Zhang, & Stepanyants, 2018). This leads to at least two interesting questions to be explored in the future: (a) What is the functional impact of that additional higher order structure on network dynamics? and (b) Given a biologically plausible model of structural plasticity, could the network reach similar higher order structure starting from both NMC- and cloud-models?

Our method of separating first-and higher order structure need not only be applied to statistical connectome models, but might also be useful for the interpretation of future dense reconstructions of brain tissue using electron microscopy (Kasthuri et al., 2015). Once a volume large enough to contain several neurons of different m-types can be reconstructed, comparing the cloud connectivity of average reconstructed neurons to the actual biological connectivity could serve as a powerful control to interpret the structure of synaptic connectivity. In particular, it can help quantify how much structure emerges from plasticity mechanisms (Zhang et al., 2018), and how much is determined by other factors, such as neuronal morphologies (Gal et al., 2017; Reimann, Horlemann, et al., 2017).

In summary, we investigated the functional impact of higher order network structure of neocortical microcircuitry. Complementing previous investigations of the relation between higher order structure and activity in simplifying recurrent neural networks (Bojanek et al., 2019; Hu et al., 2013; Recanatesi et al., 2019), we have quantified the relation in a biologically detailed model that takes into account important biological properties that shape network dynamics such as adapting, neuron-type specific synapses, and dendritic filtering. Using tools from algebraic topology and classical graph analysis, our analysis demonstrates just how many higher order structural properties are constrained by neuronal diversity within neuronal types. Our comparison between the two models suggests that the higher order network structure of cortical synaptic connectivity impacts emergent dynamics and might be a nonnegligible component of cortical function.

## METHODS

### Circuit Models

#### Neocortical microcircuit model (NMC-model).

Methods are based on a previously published model of a neocortical microcircuit of the somatosensory cortex of a two-week-old rat, here called the *NMC-model* (Markram et al., 2015). Synaptic connectivity (with apposition-based connectome/adjacency matrix AC_1_) between 31,346 neurons belonging to 55 different morphological types (m-types) was derived algorithmically starting from the appositions of dendrites and axons, and then taking into account further biological constraints such as number of synapses per connection and bouton densities (Reimann et al., 2015). Neuronal activity in the NMC-model was then simulated in the NEURON simulation environment (www.neuron.yale.edu/neuron/). Detailed information about the circuit, NEURON models, and the seven connectomes of the different statistical instantiations of the NMC-model analyzed in this study are available at www.neuron.yale.edu/neuron/ (Ramaswamy et al., 2015). Simulations and analysis were performed on an HPE SGI 8600 supercomputer (BlueBrain5).

#### Cloud-connectome.

Synaptic connectivity based on average morphologies (with cloud-based connectome/adjacency matrix CC) was computed with methods previously described by Reimann, Horlemann, et al. (2017). In brief, for each m-type *m*_*i*_ out of the 55 m-types, we computed $V_{m_{i}}^{dendrite}$(*x*, *y*) and $V_{m_{i}}^{axon}$(*x*, *y*), the mean dendrite and axon density of each m-type, based on 10 reconstructed morphologies per m-type, with a resolution of 2 *μm* × 2 *μm*. Next, we computed the convolution of axon and dendrite densities $V_{m_{i}\to m_{j}}^{\text{cloud-overlap}}$(Δ*y*, Δ*x*) = $V_{m_{i}}^{dendrite}$ * $V_{m_{j}}^{axon}$ for all combinations of m-types. This yielded a measure of the expected strength of the overlap of axon and dendrite for pairs of neurons at all potential relative soma positions. We then looked up this value for all pairs of neurons of a given combination of m-types *m*_*i*_ → *m*_*j*_, based on their locations in the NMC-model and formed a matrix of overlap strengths $O_{m_{i}\to m_{j}}^{\text{cloud}}$ (stored in a table). We next applied a transfer function *Õ* = *O*^2^, which was chosen to conserve distance-dependent connectivity from the NMC-model for most m-type combinations (Reimann, Horlemann, et al., 2017). We then normalized the matrix ${Õ}_{m_{i}\to m_{j}}^{\text{cloud}}$ to yield a matrix of connection probabilities. Finally, we turned the matrix into a connectome instance by randomly picking without replacement the same number of connections as in *AC*_*m*_*i*_→*m*_*j*__, that is with the following algorithm:

1.  Randomly pick an entry according to the connection probabilities in *Õ*.
2.  Place the connection associated with the picked entry and set its probability to 0 to avoid picking it again.
3.  Renormalize the connection probabilities to compensate.
4.  Repeat at Step 1 until the target number of connections has been placed.

The full cloud-based adjacency matrix CC was assembled from individual, randomly generated matrices for all 55 × 55 *m*_*i*_ → *m*_*j*_ combinations.

Five example connectomes CC_1–5_ for each of the seven NMC-connectomes AC_1–7_ are available at bbp.epfl.ch/nmc-portal/downloads → AVERAGE (Reimann, Horlemann, et al., 2017).

#### Cloud-model.

We implemented one of the cloud-model connectomes (CC_1_) within the existing neurons of the NMC-model, using preexisting synapses from the NMC-model. To keep physiological properties such as mean number of synapses per connection conserved, we rewired connections by changing the *source* of *netCon* in NEURON for all synapses in a connection to a new presynaptic neuron according to CC_1_, and we constrained this rewiring to connections with the same presynaptic m-type in the cloud- as in the NMC-model. If there were less connections of a *m*_*i*_ → *m*_*j*_ combination than required by CC_1_, we duplicated connections and their synapses. As some neurons receive input in CC_1_ from neurons that they did not receive input in in AC_1_, some connections could not be implemented. This was a particular problem for inhibitory connections, and we therefore implemented CC_1_ only for excitatory neurons. This resulted in a connectivity matrix CC_1*_ that uses CC_1_ for excitatory connections (with a 0.12% loss of connections), and conserved connectivity AC_1_ for inhibitory connections.

#### NMC*-model.

To ensure that any changes in emergent activity were not due to the 0.12% of missing connections, or to a shuffling of path lengths (shuffled delays of action potential propagation from soma to synapse), we created a control circuit in which we randomly removed exactly the same number of connections per m-type combination (0.12%) from the NMC-model as could not be implemented in the cloud-model, that is,

$$\bar{{\text{AC}}_{1*,m_{i}\to m_{j}}}=\bar{{\text{CC}}_{1*,m_{i}\to m_{j}}}.$$ (1)

We then shuffled the path length parameter between connections with the same presynaptic m-type for each postsynaptic neuron (only for excitatory neurons). This recreated the effect of potentially biologically implausible action potential delays in the cloud-model.

### Simulation

#### Spontaneous activity.

Simulation methods are identical to methods described by Markram et al. (2015): To simulate spontaneous activity, neurons were injected with a depolarizing somatic current, with currents expressed as a percentage of first spike threshold for each neuron (100% current used). The *U*_*SE*_ parameter for synaptic transmission of inhibitory and excitatory synapses was then differentially modulated by changing the extracellular calcium concentration [*Ca*^2+^]_*o*_. At [*Ca*^2+^]_*o*_ = 1.25 mM, the circuit was in an in vivo–like state of asynchronous activity with a global balance of excitation and inhibition. We simulated 20 trials of spontaneous activity (2 s) in the NMC-model, cloud-model, and NMC-model_cloud-control_ at five different [*Ca*^2+^]_*o*_ concentrations around the in vivo–like state at [*Ca*^2+^]_*o*_ = 1.25 mM. We further added two trials of 2-s duration at other [*Ca*^2+^]_*o*_ concentrations to illustrate the transition from asynchronous to synchronous activity. The first second of activity was discarded, as the circuit does not reach a resting state until the second second.

#### Evoked activity.

We simulated spontaneous activity for 7 s, as described above. After 1 s (at *t* = 0 ms, as we discard the first second) we apply a thalamic stimulus through synapses of 310 VPM fibers that innervate the microcircuit. The stimulus lasts 5 s (*t* = 0 to 5 s) and is identical to a previously described stimulus (Nolte et al., 2019; Reimann, Nolte, et al., 2017) based on in vivo thalamic recordings to whisker deflection (Bale, Ince, Santagata, & Petersen, 2015). We simulated 30 trials of the same stimulus in the NMC-model, cloud-model, and NMC-model_cloud-control_ at five different [*Ca*^2+^]_*o*_ concentrations around the in vivo–like state at [*Ca*^2+^]_*o*_ = 1.25 mM.

### Analysis

#### In-degree.

Number of presynaptic connections a neuron forms with other neurons in the microcircuit.

#### Out-degree.

Number of postsynaptic connections a neuron forms with other neurons in the microcircuit.

#### Simplices.

A *simplex* is a clique of all-to-all connected neurons. Methods and definitions were adapted from Reimann, Nolte, et al. (2017). In brief, if *G* = (*V*, *E*) is a directed graph, where *V* is a set of vertices (neurons) and *E* a set of ordered pairs of vertices (directed connections between neurons), then its *directed nD-simplices* for *n* ≥ 1 are (*n* + 1)-tuples (*v*_0_, …, *v*_*n*_) of vertices such that for each 0 ≤ *i* < *j* ≤ *n*, there is an edge in *G* directed from *v*_*i*_ to *v*_*j*_. Neuron 0 (the vertex *v*_0_), the *source* of the simplex (*v*_0_, …, *v*_*n*_), receives no input from within the simplex, but innervates all neurons in the simplex (there is an edge directed from *v*_0_ to *v*_*i*_ for all 0 < *i* ≤ *n*). Neuron 1 (*v*_1_), receives input from Neuron 0, and innervates Neurons 2 (*v*_1_) to *n* (*v*_*n*_), and so forth. Neuron *n*, the *sink*, receives input from all neurons in the simplex, but does not innervate any (there is an edge directed from *v*_0_ to *v*_*i*_ for all 0 < *i* ≤ *n*). See Figure 7B4 for an illustration. Note that reciprocal connections are counted separately: an *n*-simplex in *G* is defined by the (ordered) sequence (*v*_0_, …, *v*_*n*_), but not by the underlying set of vertices (neurons). For instance (*v*_1_, *v*_2_, *v*_3_) and (*v*_2_, *v*_1_, *v*_3_) are distinct 2*D*-simplices with the same neurons. We computed simplices using *flagser* (https://github.com/luetge/flagser).

Note that to avoid redundancy, the average correlation in Figures 6ABC is for *maximal* simplices, simplices that are not part of any higher dimensional simplices (Reimann, Nolte, et al., 2017), whereas all other figures show all simplices, including ones that are fully contained within simplices of a higher dimension.

#### Higher order in-degree.

We define the *N*D-in-degree as the number of *N*D-simplices a neuron is the sink of. For 1D-simplices (a pair of connected neurons), this is simply the in-degree.

#### Simplex participation of pairs of neurons.

We define as the *N*D-participation of connections at the source or the sink of a simplex how many *N*D-simplices a connection is part of as the source (Neurons 0 and 1) or at the sink (neurons *N* − 1 and *N*).

#### Betti numbers.

In brief, Betti numbers describe the number of cavities or “holes” formed by the simplices in each dimension. Betti numbers were computed using *flagser* (https://github.com/luetge/flagser). Detailed methods are as previously described by Reimann, Nolte, et al. (2017).

#### Euler characteristic.

The alternating sum of the number of simplices in each dimension (and of nonzero Betti numbers).

#### Small-worldness.

Methods are as defined by Gal et al. (2017) and were computed using the Brain Connectivity Toolbox (Rubinov & Sporns, 2010). In brief, we first computed the characteristic path length of the network, defined as the mean shortest directed path length averaged across all pairs of mutually reachable neurons:

$$l=\frac{1}{N(N-1)}\underset{i\neq j}{\sum }l_{ij},$$ (2)

where *l*_*ij*_ denotes the length of the shortest path from neuron *i* to *j*.

The clustering coefficient of a node in a directed network, *c*_*i*_, indicating the tendency of the node’s neighbors to cluster together, was defined as the ratio of the number of existing triangles among the node and its neighbors to the number of all possible triangles (Fagiolo, 2007):

$$c_{i}=\frac{{\left(M+M^{T}\right)}_{ii}^{3}}{2(d_{i}^{\text{tot}}(d_{i}^{\text{tot}}-1)-2M_{ii}^{2})},$$ (3)

where *M* is the binary connection matrix and $d_{i}^{\text{tot}}$ the combined in- and out-degree of each neuron *i*. Finally, we defined the network-wide clustering coefficient as

$$c=\frac{1}{N}\underset{i}{\sum }c_{i}.$$ (4)

Thus, *c* = 0 indicates that there are no common neighbors, and *c* = 1 indicates that all neighbors are mutually connected. The ratio of *c*/*l* gives indication about the small-worldness of the network. We showed previously that the NMC-model has a small-world topology by comparing it to different control models (Gal et al., 2017). A smaller value of *c*/*l* for the cloud than for the NMC-model thus shows that small-worldness decreases.

#### Triplet motif counting.

Triplet motifs were counted using the netsci Python library (Gal, Perin, Markram, London, & Segev, 2019) available at https://github.com/gialdetti/netsci.

#### Firing rate.

We defined the firing rate (FR) as the average number of spikes in a time bin of size Δ*t*, divided by Δ*t*.

#### Spike-time reliability.

Spike-time reliability was quantified with a correlation-based measure (Schreiber, Fellous, Whitmer, Tiesinga, & Sejnowski, 2003). The spike times of each neuron *n* in each trial *k* (*K* = 30 trials) were convolved with a Gaussian kernel of width *σ*_*S*_ = 5 *ms* to result in filtered signals *s*(*n*, *k*; *t*) for each neuron *n* and each trial *k* (Δ*t*_*S*_ = 0.5 *ms*). For each neuron *n*, the spike-timing reliability is defined as the mean inner product between all pairs of signals divided by their magnitude:

$$r_{spike}(n)=\frac{2}{K(K-1)}\underset{k\neq l}{\sum }\frac{s(n,k;t)\cdot s(n,l;t)}{\left|s(n,k;t)|\cdot |s(n,l;t)\right|}.$$ (5)

Note that *r*_*spike*_ is thus the mean reliability over all trials *and* time. Computation of spike-time reliability is identical to a previous study (Nolte et al., 2019).

#### Correlation coefficients.

We computed peristimulus time histograms (PSTHs) for each neuron *i* to the 30 trials of the thalamic stimulus (with a bin size Δ*t* = 20 ms), and next computed the normalized covariance matrix of the PSTHs of all neurons:

$$R_{ij}=\frac{C_{ij}}{\sqrt{C_{ii}C_{jj}}}.$$ (6)

*C*_*ij*_ is the covariance of PSTHs of neurons *i* and *j*. The analysis is replicating a previous analysis by Reimann, Nolte, et al. (2017). Population-based correlation coefficients used aggregated PSTHs of the population of neurons (either subpopulation for spatial correlations, or whole circuit for correlations between trials and circuits).

## ACKNOWLEDGMENTS

We thank the Blue Brain team for developing and maintaining the microcircuit model and computational infrastructure. We thank Kathryn Hess, Idan Segev, and Eilif Muller for helpful discussions. We thank Taylor H. Newton for help in editing the manuscript. This study was supported by funding to the Blue Brain Project, a research center of the École Polytechnique Fédérale de Lausanne, from the Swiss government’s ETH Board of the Swiss Federal Institutes of Technology. E. G. was supported by the Drahi Family Foundation to Idan Segev, and a grant from the EU Horizon 2020 program (720270, Human Brain Project).

## SUPPORTING INFORMATION

Supporting information for this article is available at https://doi.org/10.1162/netn_a_00124.

## AUTHOR CONTRIBUTIONS

Max Nolte: Conceptualization; Investigation; Methodology; Software; Validation; Visualization; Writing - Original Draft; Writing - Review & Editing. Eyal Gal: Investigation. Henry Markram: Conceptualization; Supervision. Michael Wolfgang Reimann: Conceptualization; Methodology; Software; Supervision; Validation; Visualization; Writing - Review & Editing.

## FUNDING INFORMATION

The Swiss government’s ETH Board of the Swiss Federal Institutes of Technology. Eyal Gal, Drahi Family Foundation. Eyal Gal, EU Horizon 2020 Programme, Award ID: 720270. cole Polytechnique Fédérale de Lausanne (http://dx.doi.org/10.13039/501100001703).

## TECHNICAL TERMS

Synaptic connectivity: The connections formed between neurons via chemical synapses.
Motif: A specific subnetwork of neurons that can repeatedly be found across the whole network.
Neocortical microcircuit: A local collection of highly interconnected neurons in the neocortex that stretches across six layers.
Dendritic and axonal cloud: The average dendritic and axonal surface area of neurons of the same morphological type.
Axo-dendritic apposition: A spatial location in which axons and dendrites of two different neurons are close enough to each other to form a synapse.
In- (and out-) degree: The number of incoming and outgoing connections of a neuron.
(Micro-) connectome: The complete set of synaptic connections formed between a collection of neurons.
Directed simplex: A specific motif of all-to-all connected neurons with directed connectivity between an input and an output neuron.
Neuronal morphology: The shape of a neuron, particularly of its dendrites and axons.
Directed network: A network of vertices (neurons) connected by directed edges (synapses) that transmit information in a specific direction.
