# Supplementary material for: Impact of higher order network structure on emergent cortical activity
Source: Netw Neurosci. 2020 Mar 1;4(1):292–314. doi: 10.1162/netn_a_00124 (PMC7069066; doi:10.1162/netn_a_00124)
Supplement: Supplementary file 1 [file netn-04-292-s001.pdf]

Nolte, M., Gal, E., Markram, H., & Reimann, M. W. (2020). Supporting information for "Impact of higher order network structure on emergent cortical activity." *Network Neuroscience*, 4(1), 292–314. https://doi.org/10.1162/netn_a_00124

SUPPLEMENTARY FIGURES (S1 TO S7)

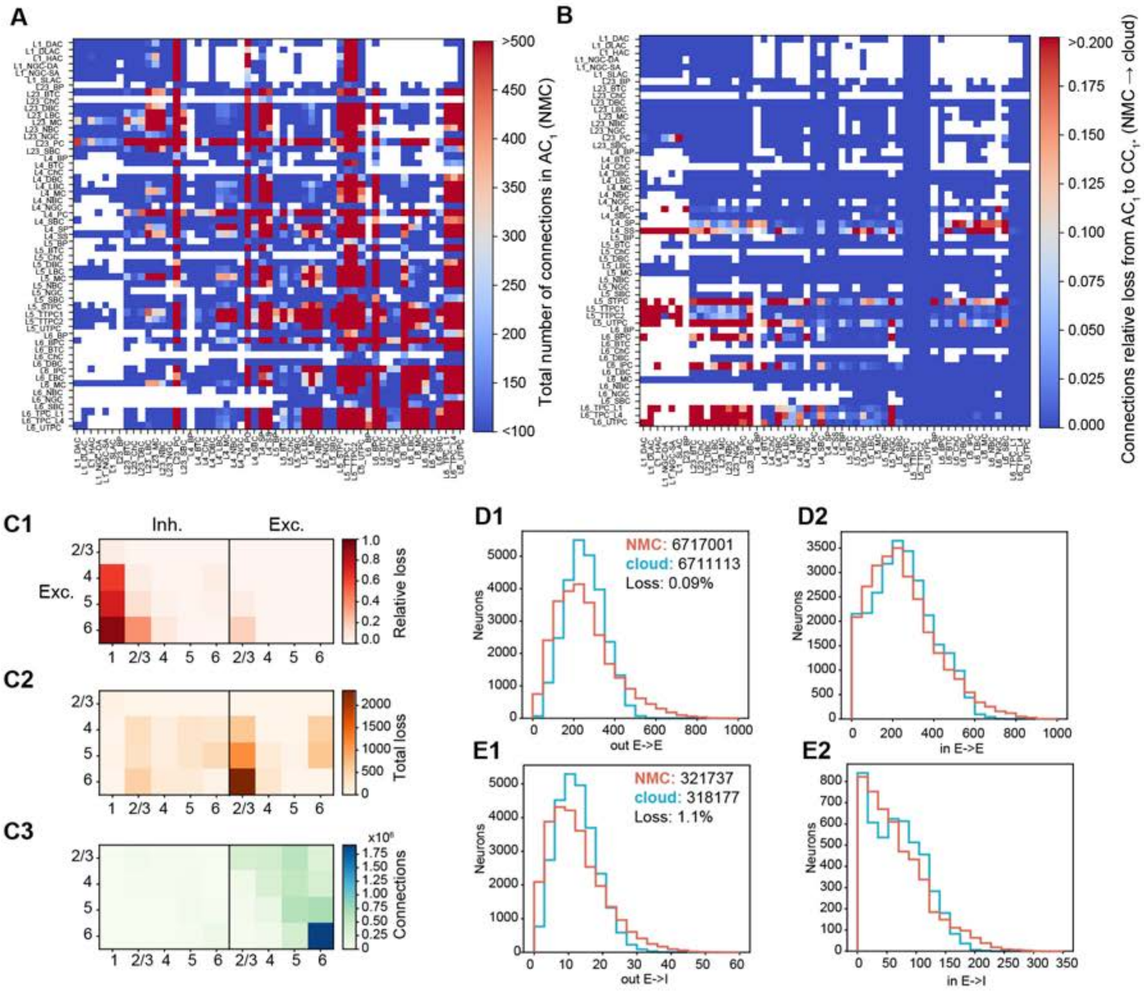

Figure S1. Macro-connectome of NMC- and cloud-models. (A) Total number of connections between m-types in NMC-model. (B) Relative loss of connections from NMC- to cloud-model between each combination of pre- and postsynaptic m-types. (C1) Relative loss of connections from NMC- to cloud-model between layers and excitatory and inhibitory neurons. (C2) Total loss of connections from NMC- to cloud-model between layers and excitatory and inhibitory neurons. (C3) Total number of connections in NMC-model between layers and excitatory and inhibitory neurons. (D1) Out-degree of excitatory neurons counting only excitatory (E) connections. Numbers describe total numbers of E-to-E connections in NMC- and cloud-models. (D2) In-degree of excitatory neurons counting only excitatory connections. (E1) Out-degree of excitatory neurons counting only connections formed with postsynaptic inhibitory (I) neurons. Numbers describe total numbers of E-to-I connections in NMC- and cloud-models. (E2) In-degree of inhibitory neurons counting only excitatory connections.

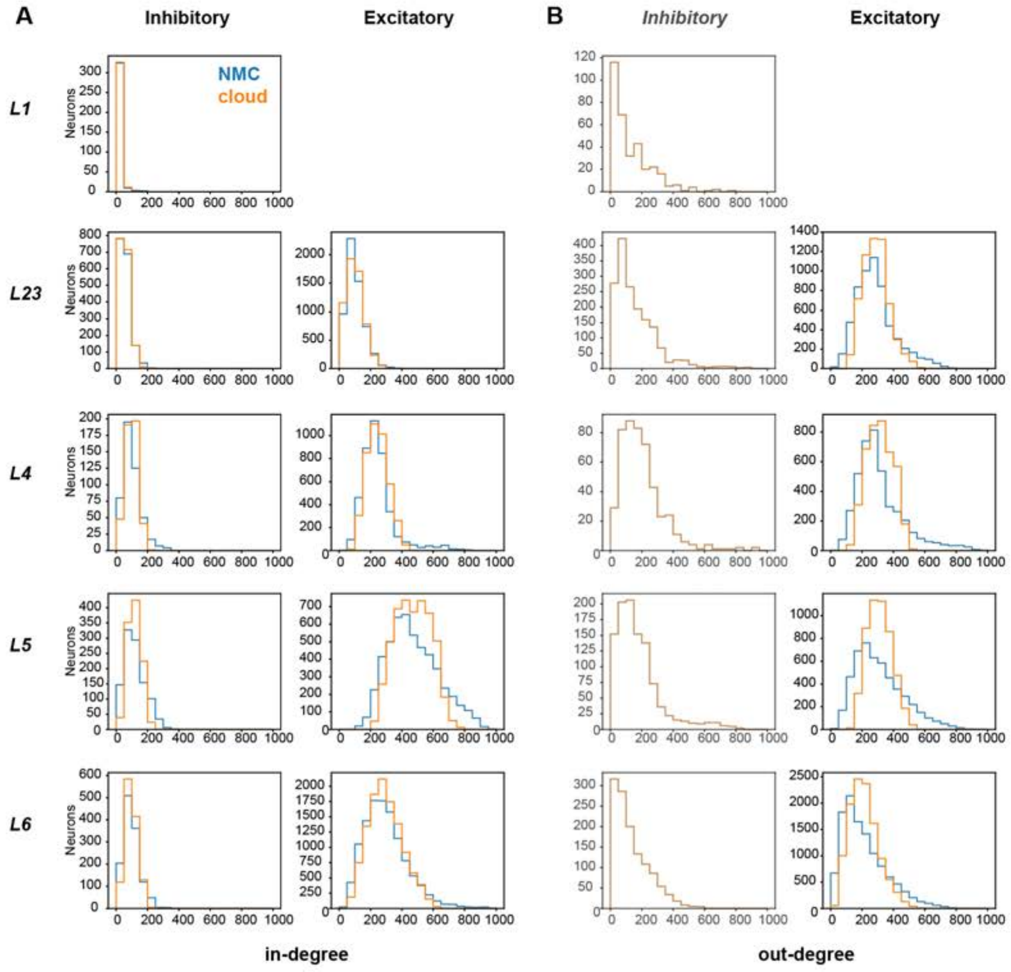

749 **Figure S2. In- and out-degrees across layers.** (A) In-degrees of neurons in NMC- and cloud-models, by layer and excitatory/inhibitory sub-type. (B)
750 Out-degrees of neurons in NMC- and cloud-models, by layer and excitatory/inhibitory sub-type.

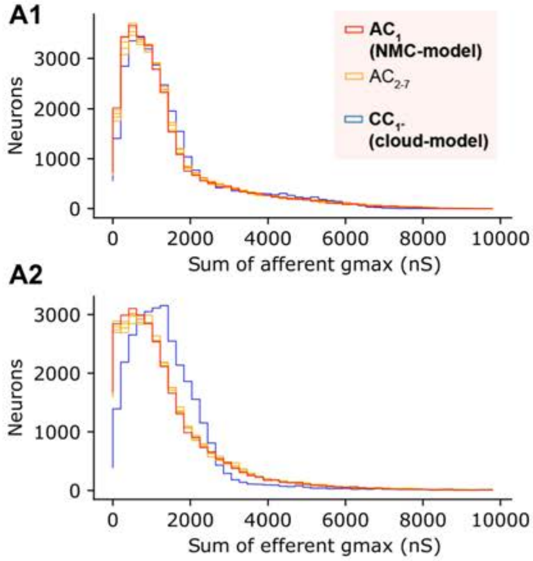

Figure S3. Sum of synaptic strengths of incoming and outgoing connections. (A) Sum of the maximum synaptic conductance (gmax) of all afferent synapses of a neuron. (B) Sum of the maximum synaptic conductance (gmax) of all efferent synapses of a neuron.

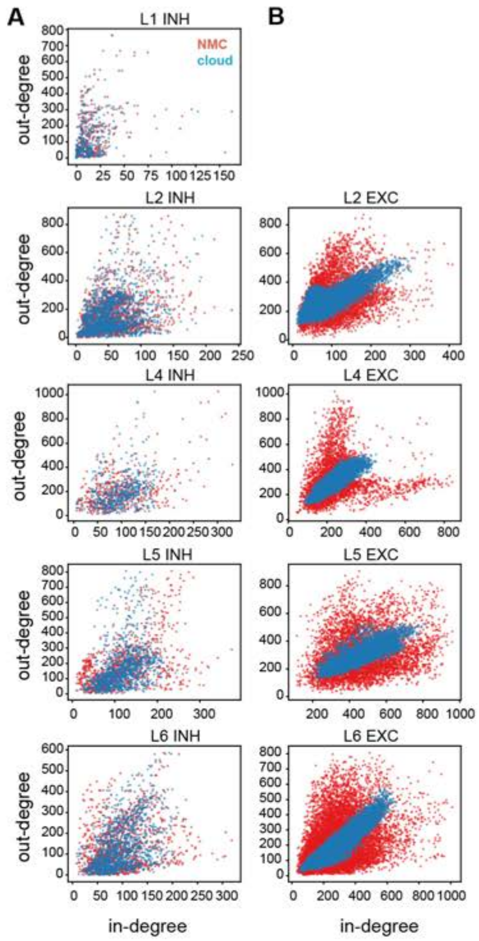

753 **Figure S4. In- and out-degree correlations.** (A) Correlations between in- and out-degree for all inhibitory neurons in NMC- and cloud-models, sorted by
754 layer. (B) Correlations between in- and out-degree for all excitatory neurons in NMC- and cloud-models, sorted by layer.

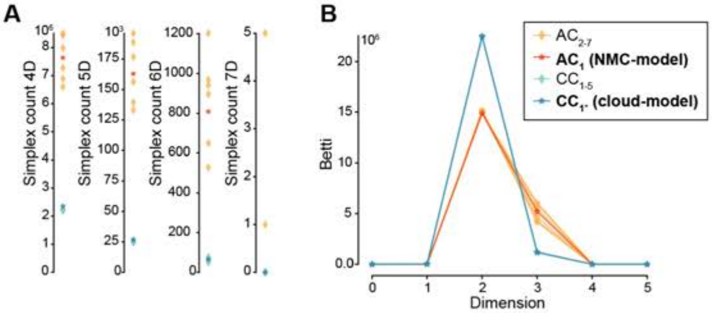

Figure S5. High-dimensional simplices and Betti-numbers. (A) Number of simplices of dimensions 4, 5, 6 and 7 across connectomes. Same legend as B. (B) Betti-numbers across connectomes.

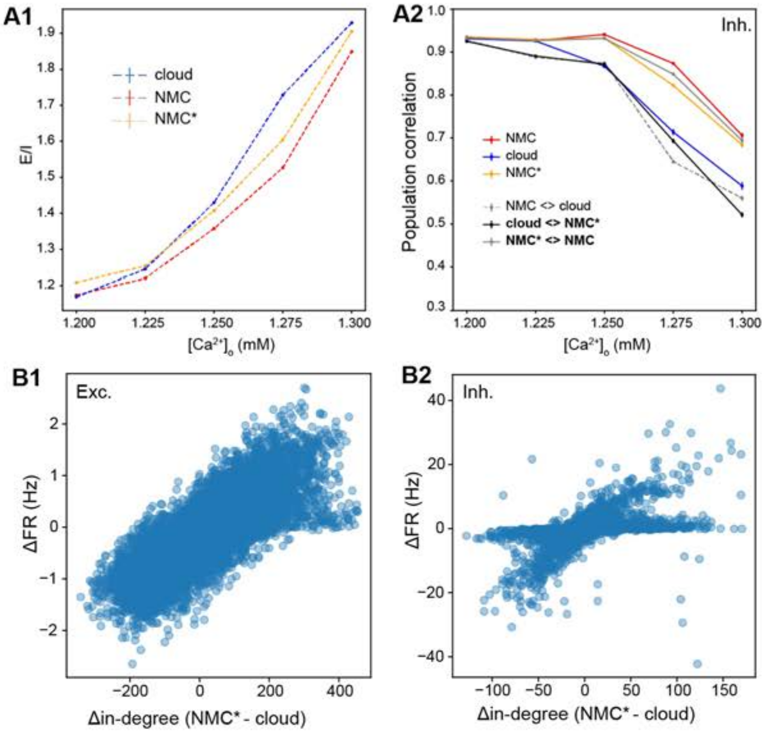

Figure S6. Firing rates and in-degree. (A1) Total spike count of excitatory neurons divided by the spike count of inhibitory neurons. Mean of 30 trials of 6000 ms, error bars indicate standard error of the mean. (A2) Average correlation coefficient between inhibitory population PSTHs ($\Delta t = 5$ ms) for 30 trials. Mean of $30 \times (30 - 1)/2 = 435$ combinations for same model, and mean of $30 \times (30 + 1)/2 = 465$ combinations between models. (B1) Difference in firing rate during six seconds of evoked activity between NMC*-model and cloud-model vs. difference in in-degree. Blue dots indicate values for individual excitatory neurons. (B2) As B1, but for inhibitory neurons.

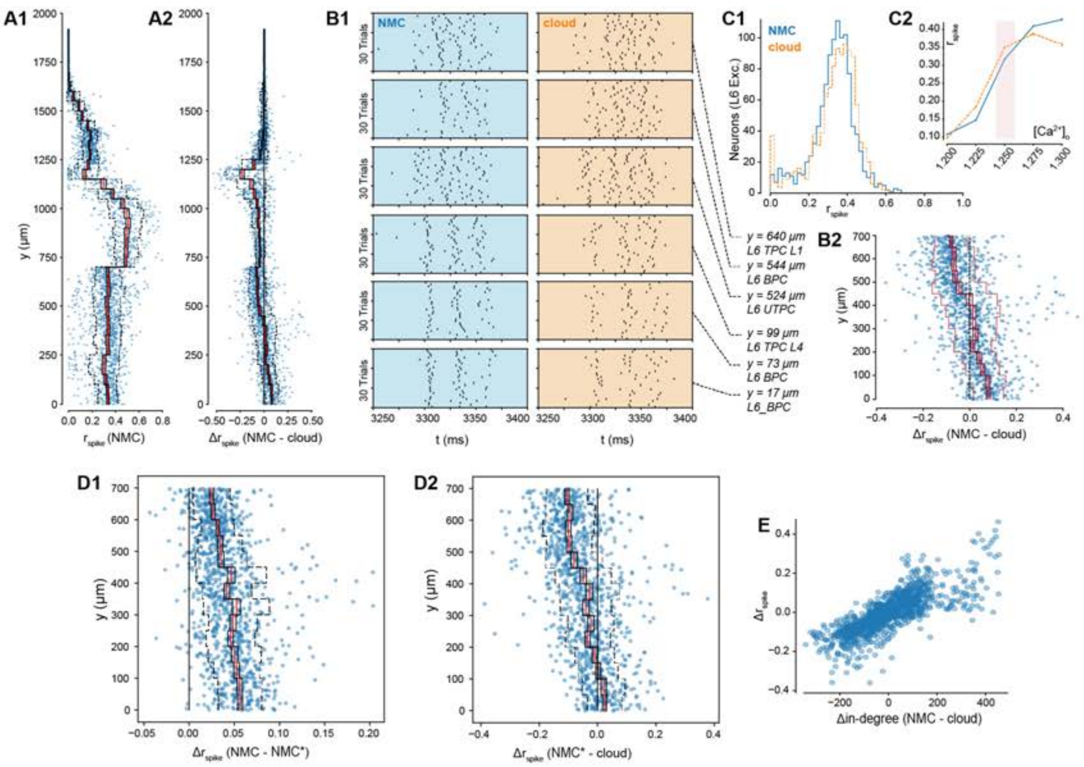

Figure S7. Spike-time reliability. (A1) Spike-time reliability r_{spike} of neurons across layers in NMC-model, at $[Ca^{2+}]_o = 1.25$ mM. (A2) Difference in r_{spike} of neurons across layers in NMC- and cloud-models at $[Ca^{2+}]_o = 1.25$ mM. (B1) Raster plot of six selected layer six (L6) excitatory neurons during 30 trials of evoked activity, at $[Ca^{2+}]_o = 1.25$ mM. (B2) Difference in spike-time reliability of L6 excitatory neurons during evoked activity at $[Ca^{2+}]_o = 1.25$ mM between NMC-model and cloud-model. Blue dots indicate values for individual neurons, ordered along their soma positions with respect to the y-axis (cortical depth). Lines indicate mean (bright red), standard-error (black), and standard deviation (dashed). (C1) Spike-time reliability r_{spike} values for individual L6 excitatory neurons for NMC- and cloud-models at $[Ca^{2+}]_o = 1.25$ mM. (C2) Mean of C1 at various $[Ca^{2+}]_o$ levels. (D1) As B2, but between NMC- and NMC*-models. (D2) As B2, but between NMC*- and cloud-models. (E) Difference in Firing rate vs. spike-time reliability between NMC- and cloud-models at $[Ca^{2+}]_o = 1.25$ mM.
